# Epidemiological insights into thoracic and lumbar sympathectomies in Brazil: a comparative analysis of open versus video-assisted procedures

**DOI:** 10.1590/acb397124

**Published:** 2024-10-07

**Authors:** Beatriz de Holanda Name, Christiane Nazareth Silva, Marcelo Henrique Ribeiro Amoroso, Eduardo Mujica Pedrosa, Daniel Amaro Sousa

**Affiliations:** 1 Centro Universitário de Brasília – Brasília (DF) – Brazil.; 2 Centro Universitário do Planalto Central Apparecido dos Santos – Brasília (DF) – Brazil.

**Keywords:** Brazil, Health Care Costs, Hospitalization, Sympathectomy, Video-Assisted Surgery

## Abstract

**Purpose:**

To investigate the profile of hospital admissions for sympathectomies performed in the Brazilian Unified Health System (SUS), comparing open and video-assisted techniques.

**Methods:**

Data on sympathectomies were collected from the SUS Department of Informatics (DATASUS), recorded between 2014 and 2023. The data were tabulated, and descriptive statistics and correlation analyses were performed.

**Results:**

There was reduction in the number of admissions for all sympathectomies during the analyzed period. The use of video-assisted surgeries was higher than that of open surgeries for thoracic procedures, but lower for lumbar procedures. The costs of hospital admission for the procedures were similar, although the length of stay and mortality associated with open surgeries were higher, both in thoracic and lumbar sympathectomies.

**Conclusions:**

The collected data were not individualized, preventing follow-up. Additionally, the study did not account for procedures performed in the private healthcare system. Despite its limitations, this study provides an overview of sympathectomies in Brazil, indicating that, although open sympathectomies are potentially more disadvantageous, they are still widely performed, especially for lumbar procedures.

## Introduction

Hyperhidrosis is a dermatological condition characterized by excessive sweating, which can occur either generally or more commonly in focal areas such as the axillae, palms, and soles^1^. By its nature, hyperhidrosis has a detrimental effect on social, professional, and psychological domains, resulting in a considerable decrease in the quality of life for affected individuals^2^.

Initial treatment focuses on topical approaches, such as the local external use of aluminum salts or anticholinergics, aiming at a temporary reduction in sweating^3^. Botulinum toxin is also used for this purpose, providing a longer-lasting effect. Oral anticholinergics, utilized as a second-line therapy, are effective but frequently cause a substantial number of side effects. Additionally, minimally invasive techniques such as iontophoresis or the use of lasers and microwaves for localized destruction of sweat glands are also considered^4^.

In failure or unavailability of the previous options, sympathectomy remains an option. This procedure involves the destruction of sympathetic ganglia connected to the sweat glands, through excision, ablation, or clipping. For axillary and palmar hyperhidrosis, the procedure is performed via thoracic approach, usually involving bilateral sectioning of the T3 ganglia^5^. For plantar hyperhidrosis, it is performed via lumbar approach, interrupting the L2-L4 ganglia^6^. Both procedures can be performed through an open approach; however, with the advent of video-assisted surgery, hospital stay and recovery time are reduced, although this modality may potentially require more advanced technology and higher costs^7^.

Epidemiological studies are essential for evaluating different surgical techniques, encouraging the adoption of more effective and safer practices. Thus, the present study aimed to analyze the profile of hospital admissions for thoracic and lumbar sympathectomies recorded in the Brazilian Unified Health System (SUS) from 2014 to 2023, comparing open and video-assisted techniques.

## Methods

This retrospective cross-sectional study was conducted using data collected from the public records of the SUS Hospital Information System, corresponding to the period from 2014 to 2023, available in the SUS Department of Informatics (DATASUS), under the Ministry of Health of Brazil. For this purpose, data were selected from the group of procedures classified as “sympathectomies,” using the following variables: region, state, year of service, regime, admission, total cost, average length of stay, deaths, and mortality rate. General population data, as well as data disaggregated by region and state, were retrieved from the Brazilian Institute of Geography and Statistics (IBGE). Costs were expressed in Brazilian real (BRL). The data were tabulated using GraphPad Prism version 10.1.2, and regression lines were created using the same software. The association between groups was assessed using the χ^2^ test with the MedCalc software, considering a significance level of 5% (< 0.05).

This study was exempt from ethical committee review, as it involves research using aggregated databases without the possibility of individual identification, in accordance with the Brazilian National Health Council Resolution No. 510, 2016, item V.

## Results

**Figure 1:**
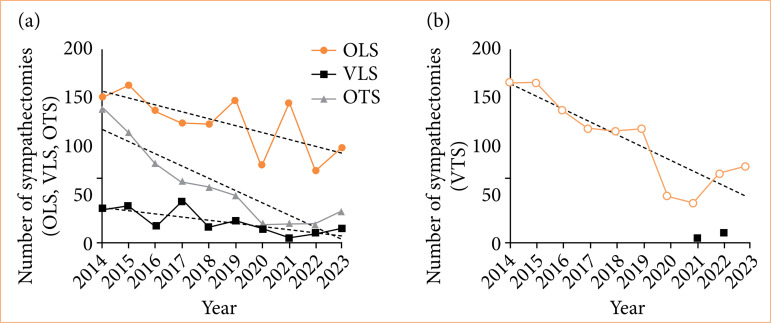
Annual evolution of the absolute number of sympathectomies by modality. **(a)** Open lumbar sympathectomy (OLS), open thoracic sympathectomy (OTS), and video-assisted lumbar sympathectomy (VLS); **(b)** video-assisted thoracic sympathectomy (VTS). Dashed lines indicate linear regression lines.

Source: Elaborated by the authors based on data collected from the Unified Health System (SUS) Hospital Information System/SUS Department of Informatics.

From 2014 to 2023 in Brazil, a total of 9,898 sympathectomies was reported, with an average of 990 per year. Considering the total number of surgical admissions during the period (46,931,411), sympathectomies accounted for only 0.02% of this total. When divided by procedure type, 1,417 were lumbar sympathectomies, of which 85.2% were open surgery and 14.8% were video-assisted. Among the 8,481 thoracic sympathectomies, 93.1% were performed using video-assisted surgery, while only 6.9% were open surgery. Based on the plotted linear regression line for the number of sympathectomies by year divided by modality (Fig. 1), a downward trend is observed for all procedures, as indicated by the decreasing slopes.

Regarding the geographical distribution of the procedures in Brazil, considering the population as of mid-2019 according to data of the IBGE data, the South region had the highest number of sympathectomies per capita (10.01 per million for lumbar and 95.54 per million for thoracic), followed by the Southeast region (8.78 per million for lumbar and 43.5 per million for thoracic). Combined, the South and Southeast regions account for 78% of video-assisted sympathectomies and 78.1% of open sympathectomies. Notably, the proportion of video-assisted open surgeries varied by region, being 6.6:1 in the Brazilian South region, 6.5:1 in the Central-West region, 4:1 in the Northeast region, 3.6:1 in the Southeast region, and 2.71:1 in the North region.

In absolute numbers, São Paulo is the Brazilian state with the highest number of sympathectomy admissions (31.67% or 69 admissions per million inhabitants), followed by Rio Grande do Sul (14.2% or 67 admissions per million inhabitants), Paraná (10.9% or 63 admissions per million inhabitants), and Minas Gerais (9.9% or 66 admissions per million inhabitants). These four states accounted for two-thirds of the total sympathectomies reported during the period. Among these states, the ratio of video-assisted to open sympathectomies was 7.9:1 in Paraná, 7.4:1 in Minas Gerais, 5.2:1 in Rio Grande do Sul, and 2.95:1 in São Paulo.

Regarding the costs associated with sympathectomy admissions, a total of R$ 11,062,524.10 was spent during 2014 to 2023 period, with 85% allocated to thoracic sympathectomies and 15% to lumbar procedures. Out of the amount allocated to thoracic procedures, 85.2% was for open surgeries, while for lumbar procedures 93.5% was for video-assisted surgeries. The average costs per procedure were similar, calculated by dividing the total costs by the number of procedures. The mean cost for open lumbar sympathectomy was R$ 1,158; for video-assisted lumbar sympathectomy, it was R$ 1,091; for open thoracic sympathectomy, it was R$ 1,044; and for video-assisted thoracic sympathectomy, it was R$ 1,117. The difference between open and video-assisted procedures was statistically non-significant for both lumbar and thoracic approaches (*p* = 0.1577 and *p* = 0.1163, respectively).

**Figure 2:**
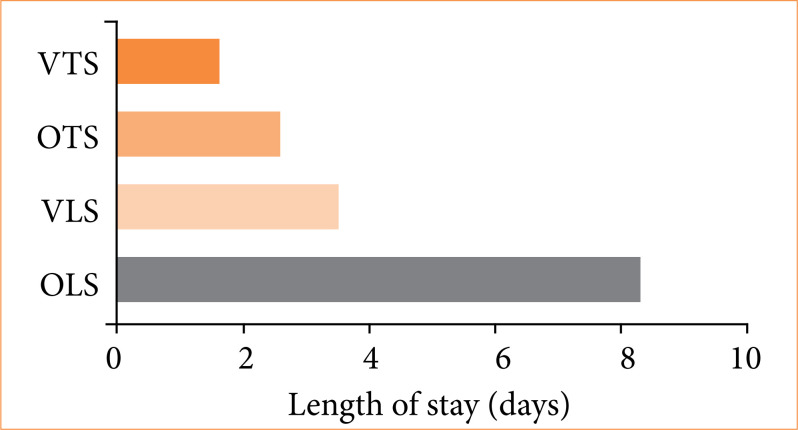
Average length of stay by procedure.

VTS: video-assisted thoracic sympathectomy; OTS: open thoracic sympathectomy; VLS: video-assisted lumbar sympathectomy; OLS: open lumbar sympathectomy. Source: Elaborated by the authors based on data collected from the Unified Health System (SUS) Hospital Information System/SUS Department of Informatics.

The average length of stay was longer for open surgeries than for video-assisted procedures for both lumbar and thoracic sympathectomies. For lumbar sympathectomy, the average stay was 5.9 days, with 8.3 days for open surgeries and 3.5 days for video-assisted procedures. For thoracic sympathectomy, the average was 2.1 days, with 2.6 days for open surgeries and 1.6 day for video-assisted procedures (Fig. 2). The states with the longest average stay for all procedures were Acre, Piauí, Rio de Janeiro, and Maranhão (average 7.2 days), while those with the shortest stay were Rio Grande do Norte, Paraíba, Alagoas, and Sergipe (average 1.32 days).

Regarding mortality, only one death was reported for video-assisted lumbar sympathectomy, resulting in a mortality rate of 0.47%. For open lumbar sympathectomy, 17 deaths were reported, with a three times higher mortality rate (1.4%). For thoracic sympathectomy, two deaths were reported for open surgery, with a mortality rate of 0.33%, while six deaths were reported for video-assisted surgery, with a four times lower mortality rate of 0.08%. Geographically, out of the 26 total deaths reported, 42.3% occurred in São Paulo (11 deaths), 23% in Minas Gerais (six deaths), and 11% in Goiás (three deaths). The remaining 23% were reported in Santa Catarina, with two deaths, and Rio de Janeiro, Sergipe, Paraná, and the Federal District, with one death each. No deaths were reported in other states.

In absolute numbers, the number of deaths was similar between the public (five deaths) and private (four deaths) sectors. However, it is important to note that, in 65.4% of cases, this information was not recorded. The overall mortality rate for all sympathectomies, including both sectors and incomplete data, was calculated as 0.26%. When specified by each sector alone, the mortality rate was 0.32% in the public sector and 0.30% in the private sector.

## Discussion

The number of sympathectomies in Brazil has decreased over the past decade. This decline can generally be attributed to the emergence and dissemination of new therapeutic options, such as new antiperspirant and anticholinergic drugs, botulinum toxin, iontophoresis, and microneedling^8^. Thus, considering that surgical intervention should be postponed as the last resort for hyperhidrosis treatment^9^, this reduction should be expected. The more pronounced decrease in 2020 can be attributed to the COVID-19 pandemic, which affected not only sympathectomies^10^, but also most surgical procedures in Brazil, including the suspension of elective surgeries^11^.

Regarding the spatial distribution of hospitalizations for sympathectomies, it is noteworthy that, despite accounting for just over half of the population (56%), more than 78% of sympathectomies were performed in the Southern and Southeastern regions. This proportion differs significantly from the number of hospitalizations for all surgical procedures in the period also recorded in the DATASUS system, which tends to be proportional to the population size in each region (58% in the southern and southeastern regions).

Thoracic sympathectomies are performed in greater proportion than lumbar sympathectomies (6:1). Brazilian prevalence studies on hyperhidrosis conducted in limited population groups indicate a predominance of this condition in the axillary and palmar regions^12^^–^ 14, which primarily require thoracic sympathectomy. Additionally, although highly effective in treating plantar hyperhidrosis, which is less common in the population, lumbar sympathectomy is associated with the risk of sexual dysfunction in men due to retrograde ejaculation^15^, constituting an important contraindication factor for this procedure.

Video-assisted surgery is prevalent in thoracic sympathectomy, and, although therapeutic outcomes are equivalent, it should be prioritized due to lower morbidity rates, shorter hospital stays, and faster return to activities compared to open surgeries^16^^,^ 17. However, in lumbar sympathectomy, open surgery is more prevalent (85% of cases). Considering that this procedure is associated with a higher number of perioperative and postoperative complications^15^ and that longer hospital stays are observed in open surgeries in this study, it is advisable to prioritize video-assisted surgeries in lumbar sympathectomies as well, especially in the Southeast region, particularly in the state of São Paulo, where there tends to be not only a higher number of sympathectomies, but also a closer proportion between the number of video-assisted and open surgeries.

In both thoracic and lumbar sympathectomies, there was no significant difference in the stay costs between video-assisted and open surgeries. The literature in the field points out cost as the main disadvantage of video-assisted procedures^18^. However, since the cost data relate to the total amounts spent on hospitalizations and not just the surgical procedure, it is possible that costs associated with other complications, which are probably more frequent in open surgeries, may account for the equalization of values.

When analyzing the profile of deaths associated with hospitalizations for sympathectomies, reduced mortality was observed in video-assisted surgeries (three times lower in lumbar and four times lower in thoracic procedures). It is important to note that, due to the characteristics of the databases, it is not possible to distinguish deaths that occurred perioperatively or postoperatively. There are few reports in the literature of deaths directly associated with surgical procedures^19^, but various complications have been reported^20^^,^ 21. Therefore, further studies are needed to detail the morbidity and mortality associated with these techniques.

The use of data from the DATASUS platform prevents the follow-up and individualization of cases, ignores procedures performed by the private health system, and presents limited data^22^. By working with this source of information, the present study has many of these limitations; however, the correlations established here may be relevant for formulating hypotheses for more in-depth research that could provide a better understanding of surgical treatments for severe hyperhidrosis through sympathectomies, thus contributing to the establishment of more effective and safer therapies for this condition.

## Conclusion

The number of hospitalizations for sympathectomies has decreased in Brazil over the last 10 years, possibly due to the introduction of new treatment options for hyperhidrosis. Nevertheless, a significant number of procedures are still being performed. This study demonstrated that thoracic sympathectomies are more frequently performed, primarily using video-assisted surgery. In contrast, lumbar sympathectomies are predominantly performed as open surgeries. For all sympathectomies, open procedures presented higher mortality rates and longer hospital stays, despite similar hospitalization costs.

As the most comprehensive study on this topic in Brazil, despite its inherent limitations, this research suggests that healthcare professionals should be encouraged to adopt video-assisted sympathectomies for treating severe hyperhidrosis when indicated.

## Data Availability

The data will be available upon request.
